# A prospective study on the application of HINTS in distinguishing the localization of acute vestibular syndrome

**DOI:** 10.1186/s12883-022-02904-x

**Published:** 2022-10-05

**Authors:** Tao Qiu, Xiaoyan Dai, Xiaoya Xu, Guiqin Zhang, Linming Huang, Qingping Gong

**Affiliations:** 1 Department of Neurology, Zigong First People’s Hospital, No. 42, shangyihao 1st branch road, Ziliujing, Zigong, 643000 Sichuan China; 2 Outpatient department, Zigong First People’s Hospital, No. 42, Shangyihao 1st Branch Road, Ziliujing District, Zigong, 643000 Sichuan China

**Keywords:** Retrospective study, Acute vestibular syndrome (AVS), Central AVS, Peripheral AVS, HINTS

## Abstract

**Background:**

Acute vestibular syndrome (AVS) is a common clinical syndrome in neurology clinics and emergency department. Canonical standard for AVS diagnosis requires the presence of persistent vertigo for more than 24 h. HINTS (head impulse-nystagmus-test of skew) is an emerging scheme in the diagnosis of AVS. In this prospective study, we evaluated the specificity and sensitivity of HINTS in distinguishing between central and peripheral AVS.

**Methods:**

A cohort of 239 cases with complete clinical record was recruited in the study. All patients completed emergency brain CT examination to exclude hemorrhagic stroke. HINTS examination was conducted to distinguish between central AVS and peripheral AVS, and all patients completed head MRI, BAEP and vestibular function examinations within one week. Patients diagnosed as central AVS were subject to angiography (CTA/MRA/DSA), and patients with peripheral AVS were considered for a 3-month follow-up to correct the initial diagnosis.

**Results:**

Patients with central AVS were associated with an elder age, higher incidences of hypertension, atrial fibrillation, family history of stroke and previous history of stroke. Posterior circulation cerebral infarction, vestibular migraine and cerebellitis were the dominant diseases associated with central AVS. The sensitivities of HIT, GE, and TS in the diagnosis of central AVS were 73.5%, 61.2%, and 26.5%, and the specificities were 97.9%, 92.6%, and 93.2% respectively.

**Conclusions:**

The sensitivity of HINTS for central AVS diagnosis is 89.8% and the specificity is 84.2%. HINTS is an easy-to-operate, low-cost, high-sensitivity and specific examination technique, which is practical in neurology outpatient clinics and emergency departments.

## Introduction

Acute vertigo is a very common clinical syndrome, with an approximate incidence of 5–10% in neurology clinic [1], and an incidence of 6.7% in hospitalized cases [2]. Neurologists and emergency physicians must quickly distinguish between vertigo caused by central nervous system disorders (such as cerebellar stroke) and vertigo caused by peripheral vestibular end-organ disorders (such as vestibular neuritis), so as not to miss the best time for treatment strategies such as thrombolysis or mechanical thrombectomy. Several multicenter studies have indicated that the prevalence of central vertigo in all emergency department patients ranges from 3.2 to 12.5 percent [2–5].

Acute vestibular syndrome (AVS) is a class of clinical syndromes characterized by acute onset, persistent vertigo/dizziness, which can last for several days to several weeks and is associated with the progressive vestibular system dysfunction [6]. Canonical standard for AVS diagnosis requires the presence of persistent vertigo for more than 24 h [7]. The emerging strategies such as intravenous thrombolysis and interventional therapy could effectively ameliorate the AVS symptoms [8, 9]; however an early diagnosis within 4.5 to 6 h after the onset is critical. Therefore, in 2014 the International Panel on Classification of Vestibular Symptoms recommends to divide AVS into broad category (> 6 h) and narrow category (> 24 h hours according to the duration of symptoms [10].

CT (Computed Tomography) and MRI (Magnetic Resonance imaging) are commonly used in the diagnosis of AVS [11], but CT suffers from a poor sensitivity for early stroke and the missed diagnosis rate is as high as 60% [12]. MRI is not usually available for emergency examinations, which restricts the access to MRI diagnosis in emergency department [13]. In addition, previous studies have shown that MRI has low sensitivity for diagnosing ischemic stroke in the posterior circulation. A previous report also indicated that the missed diagnosis rate of MRI for central vertigo is as high as 20% to 35% [14]. Furthermore, the missed diagnosis of cerebellar infarction could be associated with an eightfold increased risk of death [15].

Recent studies have revealed the clinical power of HINTS (head impulse-nystagmus-test of skew) in the diagnosis of AVS. Several reports showed that HINTs is more sensitive than neuroimaging method in distinguishing stroke from AVS in patients, with a sensitivity of 100% and a specificity of 90–94.4% [16, 17]. HINTS examination consists of three parts: (1) HIT examination; (2) spontaneous nystagmus (Nystagmus); (3) eye deviation (Test of Skew). Recent reports indicate that HINTs can effectively distinguish between AVS and other central/peripheral neurological disorders in patients [7]. For example, Chen et al. reported that the positive result in any of negative HIT, central type nystagmus, conjugate ocular torsion, and abnormal vertical smooth tracking could be used as diagnosis for central AVS, with a sensitivity of 100% and a specificity of 90% in distinguishing the central AVS from stroke [17]. Although HINTS seems to be a promising diagnosis method for AVS, the procedures and standards of HINTs need to be optimized for the best clinical application [18].

In this prospective study, we aim to evaluate how accurate HINTS could be practically applied in clinics for the diagnosis of central AVS. We also intend to assess the practicability and the effectiveness of different components of HINTs (HIT examination; spontaneous nystagmus (Nystagmus) and eye deviation (Test of Skew)) in the clinical diagnosis of central AVS.

## Materials and methods

### Study subjects recruitment

From October 2019 to January 2022, patients who were admitted to the emergency department of Zigong First People's Hospital with acute vertigo and were hospitalized were included in this study. This study was approved the Ethics Committee of Zigong First Hospital. All procedures performed in studies involving human participants were in accordance with the ethical standards of the institutional and/or national research committee and with the 1964 Helsinki declaration and its later amendments or comparable ethical standards. Inclusion criteria: Acute onset, age ≥ 18 years; Admission within 24 h of onset, with dizziness as the main manifestation; Symptoms and signs of brainstem involvement such as diplopia, facial paralysis, dysarthria, limb numbness and weakness, hemianopia, eye movement disorders, and pyramidal tract signs are absent; Brain CT examination did not show accountable lesion causing the vertigo; The patients or their relatives were informed and signed the informed consent. Exclusion criteria: A definitive diagnosis of benign paroxysmal positional vertigo; There was a clear cause of dizziness, such as hypoglycemia and fever; Poor general condition such as severe nausea, vomiting, severe neck disease, external ophthalmoplegia, and inability to cooperate with the examination; The impact of Drug and alcohol usage could not be ruled out; Obvious organ insufficiency and pregnant women; Cerebellitis was excluded by infectious viral test; Patients with autoimmune disorders or neoplastic diagnosis; Contraindications to MRI examination or refusal to perform cranial MRI examination.

### Study method

This study was a prospective study aiming to understand the practical value of HINTS examination by Chinese doctors for AVS diagnosis, with a statistical analysis of the etiology of vertigo.

#### Data collection workflow

**Fig. 1:**
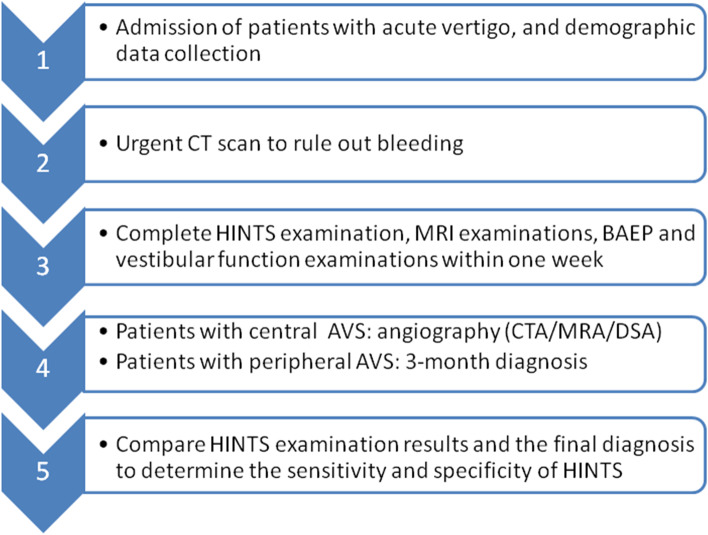
Schematic illustration of study design and data collection workflow

1. Clinical data including gender, age, hypertension, diabetes, hyperlipidemia, heart disease, overweight, lack of exercise, smoking, family history of stroke and previous history of stroke were collected. 2. All patients completed emergency brain CT examination to exclude hemorrhagic stroke. 3. Perform the HINTS examination to distinguish between central AVS and peripheral AVS. And all patients completed head MRI examinations (including T1, T2, Flaire, DWI and ADC), BAEP examination images and vestibular function examinations within one week. 4. Patients with central vertigo were considered to complete angiography (CTA/MRA/DSA), and patients with peripheral vertigo were considered for a 3-month follow-up to correct the diagnosis, and finally determine the location of the patient's vertigo. 5. The final diagnosis and  the results of HINTS examination were compared and analyzed to determine the sensitivity and specificity of HINTS. At the same time, the etiology of patients with acute vertigo was analyzed (see Fig. 1).

#### Diagnostic criteria of HINTS

**Fig. 2:**
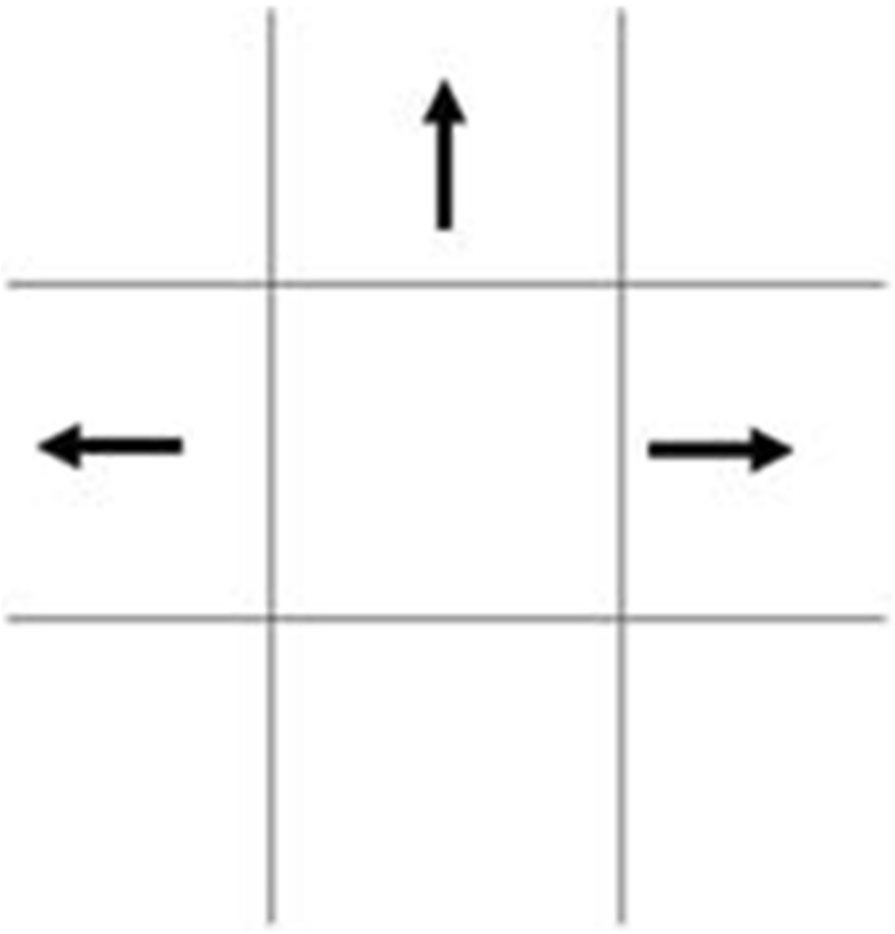
Shecmatics of the direction in Gaze-evoked nystagmus (GEN)

- 1. Head impulse test (HIT): the subject and the examiner sit opposite to each other with a distance of about 40 cm. We asked patients to fixate on the examiner’s nose. The examiner gently moved laterally the patient’s head to one side, and accelerated the head rapidly toward the center. Because of an impaired horizontal VOR, the eyes receive a suboptimal signal to stay on the target, thus requiring a visually triggered saccade to re-fixate. The horizontal head impulse test is positive when there is a significant lag, with corrective saccades, and with few exceptions, unilaterally positive head impulse test, associates frequently with peripheral vertigo. If the results are positive in AVS in both horizontal directions, it suggests central vertigo.
- 2. Gaze-evoked nystagmus (GEN), also known as direction-changing nystagmus: the subject was in an upright position; the examiner stood in front of the subject, and instructed the patient to follow the examiner's finger. During horizontal GEN examination, the patient looked at a target in a lateral eccentric position (about 30 degrees to the right and left of fixation).The examiner observed the direction of the horizontal nystagmus in left and right gaze. We identified the nystagmus as “direction changing” when the fast phase changed (See Fig. 2). If nystagmus fast phase does not change direction, regardless of eye position, then it is unidirectional.
- There are two principal types of pathological GEN in AVS patients. One vestibular type, when present with straight-ahead fixation, increases in lateral gaze (Alexander's law) in the direction of the fast phase, it has a constant slow phase velocity. A second type is the result of gaze holding failure due to a lesion of the neural integrator, thus, eccentric gaze cannot be maintained and the eyes drift back to center with re-fixation saccade toward the eccentric target (the slow phase velocity decreases exponentially). This nystagmus combination is present in central AVS patients.
- 3. Test of skew (TS): the patient is fixating on a near target during the cross-cover test and that the movement of the eyes is vertical and dissociated, on eye is high: hypertropic and one low: hypotropic. After covering one eye with the palm for 1–3 s, we covered the fellow eye in alternate fashion, and observed a vertical eye movement of the uncovered eye. The absence of vertical movement is the normal response.
- 4. HINTS judging criteria for AVS: if any of the following 3 conditions is positive in the HINTS examination, the case is considered as central AVS, otherwise it is judged as peripheral AVS: A. The horizontal head-impulse test is normal (negative); B. The direction of nystagmus changes with the direction of gaze GEN + , C. The dissociated vertical deviation (skew) of both eyes is alternately covering the eyes. TS is positive.
- 5. Diagnostic criteria for other diseases: posterior circulation transient ischemic attack (TIA) and posterior circulation cerebral infarction refer to the criteria of "China Guidelines for Diagnosis and Treatment of Acute Ischemic Stroke 2018". TIA requires vascular examination findings and symptoms, associated vascular stenosis and other related lesions. Cerebral infarction requires MRI DWI to find new posterior circulation cerebral infarction lesions that can explain the patient's symptoms. TIA is defined as transient central findings with normal DWI MRI and abnormal MRA results, and a cerebellitis diagnosis hinges on clinical signs and lumbar puncture. We emphasize the fact that acute deafness and vertigo caused by the internal auditory artery ischemia/infarction belong to the category of PCI, representing peripheral vestibular due to the anatomic lesion localization. The diagnostic criteria for vestibular migraine: the 2013 International Classification of Headache Disorders (ICHD) 3rd edition diagnostic criteria for vestibular migraine is called. Diagnostic criteria for peripheral vestibular diseases: BPPV, vestibular neuritis, Meniere's disease, labyrinthitis, sudden deafness with vertigo, all in accordance with the relevant diagnostic criteria, and consultation with an otorhinolaryngologist is required for diagnosis.

### Statistical analysis

Statistical analysis was performed using SPSS 26.0 software. The chi-square test was used to compare categorical variables between groups, and the t test was used to compare continuous variables between groups. The sensitivity, specificity, positive and negative predictive value of the two-step assessment method for the diagnosis of central vertigo were calculated, the confidence intervals (CI) were set at 95%, and the test level was set at 0.05, that is, there was a difference when *P* < 0.05.

## Results

### Clinical data of subjects

A total of 239 cases with complete data were collected in this study, including 49 cases of central AVS and 190 cases of peripheral AVS. The clinical records are summarized in Table 1.

**Table 1:** Comparison of clinical data between central and peripheral AVS cases

parameter	Central AVS (*N* = 49)	Peripheral AVS (*N* = 190)	2/t	P
age	61.47 ± 16.89	54.08 ± 10.34	2.924	**0.005**
male	24(49.0%)	84(44.2%)	0.358	0.550
female	25(51.0%)	106(55.8%)		
hypertension	24(49.0%)	25(13.2%)	30.688	**0.000**
diabetes	11(22.4%)	32(16.8%)	0.830	0.362
hyperlipidemia	26(53.1%)	81(42.6%)	1.714	0.191
atrial fibrillation	3(6.1%)	0(0.0%)	11.781	**0.001**
valvular heart disease	16(32.7%)	73(38.4%)	0.555	0.456
Obesity BMI > 30	5(10.2%)	8(4.2%)	2.721	0.099
lack of exercise	24(49.0%)	25(13.2%)	30.688	0.230
Family history of stroke	4(8.2%)	0(0.0%)	15.774	**0.000**
history of previous stroke	6(12.2%)	0(0.0%)	23.864	**0.000**

Based on the demographic data, the average age of central AVS is 7.39 years older than that of peripheral AVS, and there is a significant difference. The incidence of central AVS in males and females was similar, while for peripheral AVS, there were more female cases than males, but the difference was not statistically significant. From the perspective of risk factors, the incidences of hypertension, atrial fibrillation, family history of stroke and previous history of stroke were significantly higher in central AVS cases. While the higher incidences of hyperlipidemia, diabetes, obesity, lack of exercise in central AVS were not statistically significant.

### Disease spectrum analysis

**Fig. 3:**
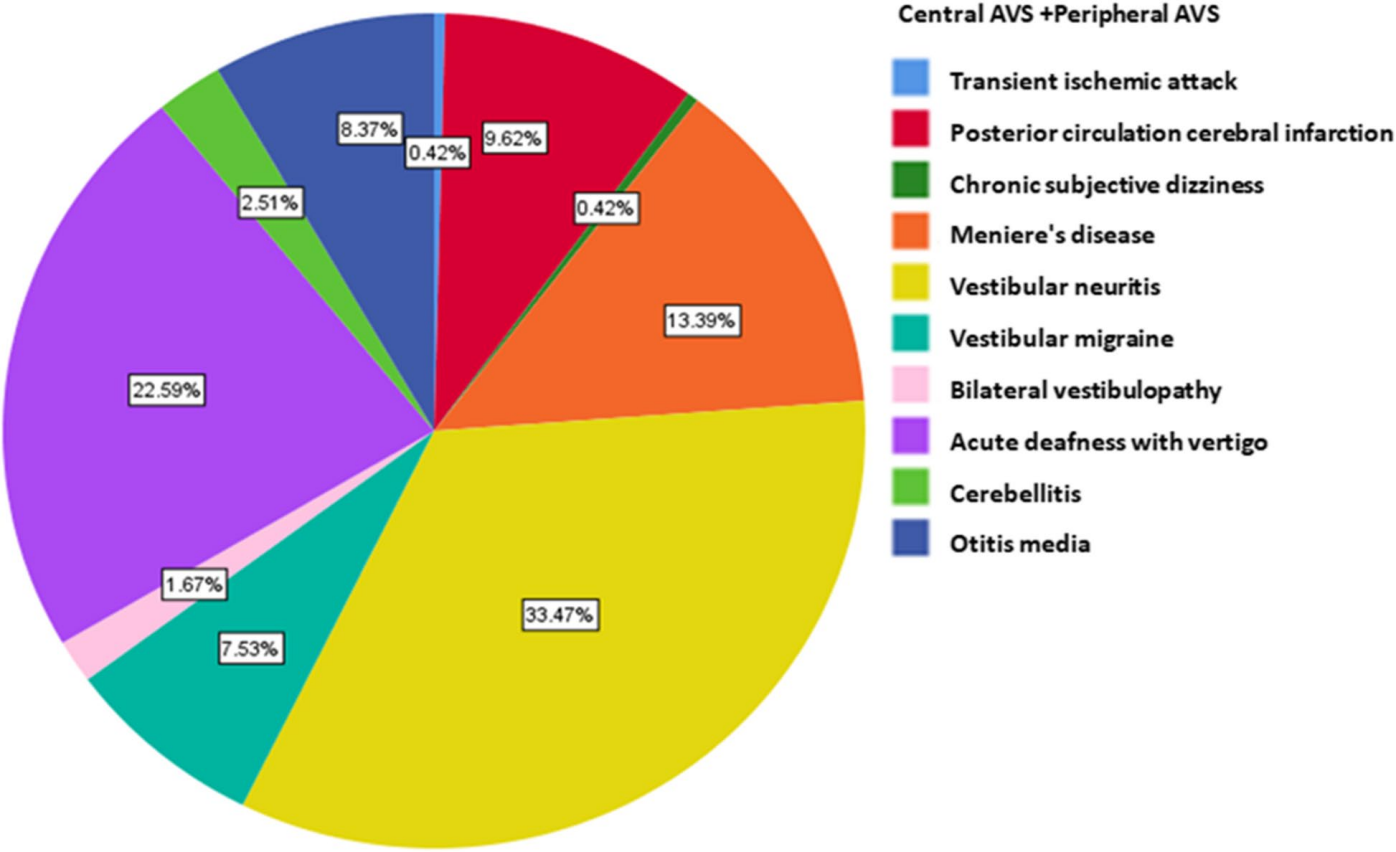
Disease spectrum of patients with central and peripheral AVS

We then examined the distribution of diseases related to central and peripheral AVS. Table 1 showed that in patients diagnosed with AVS, peripheral AVS is dominant which accounts for 79.50%, and central ACS accounts for 20.50%. Among them, the top ranked five diseases are vestibular neuronitis (33.47%,) acute deafness with vertigo (22.59%), Meniere's disease (13.39%), posterior circulation cerebral infarction (9.62%), and otitis media (8.37%) (Fig. 3).

**Fig. 4:**
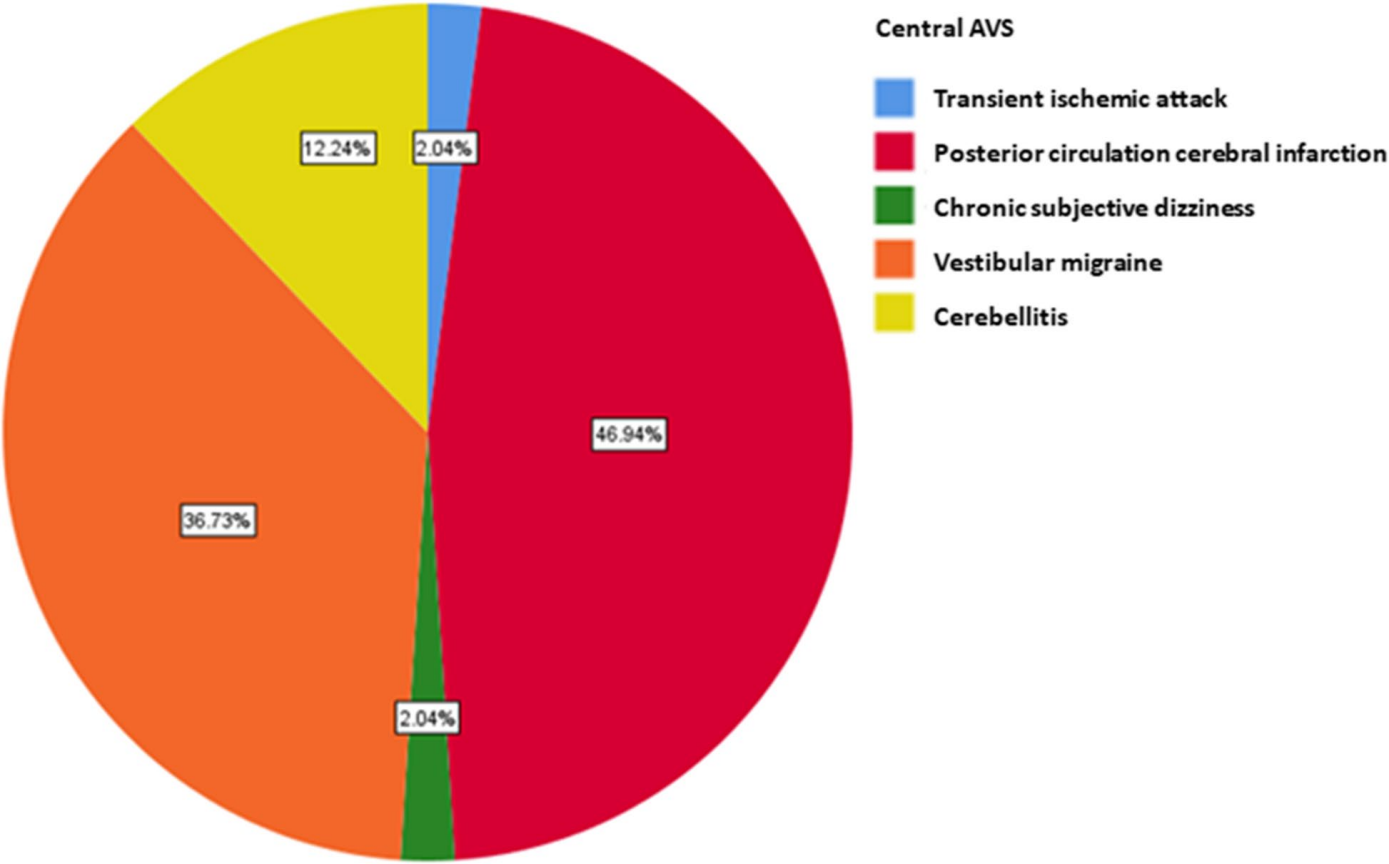
Disease spectrum of patients with central AVS

**Fig. 5:**
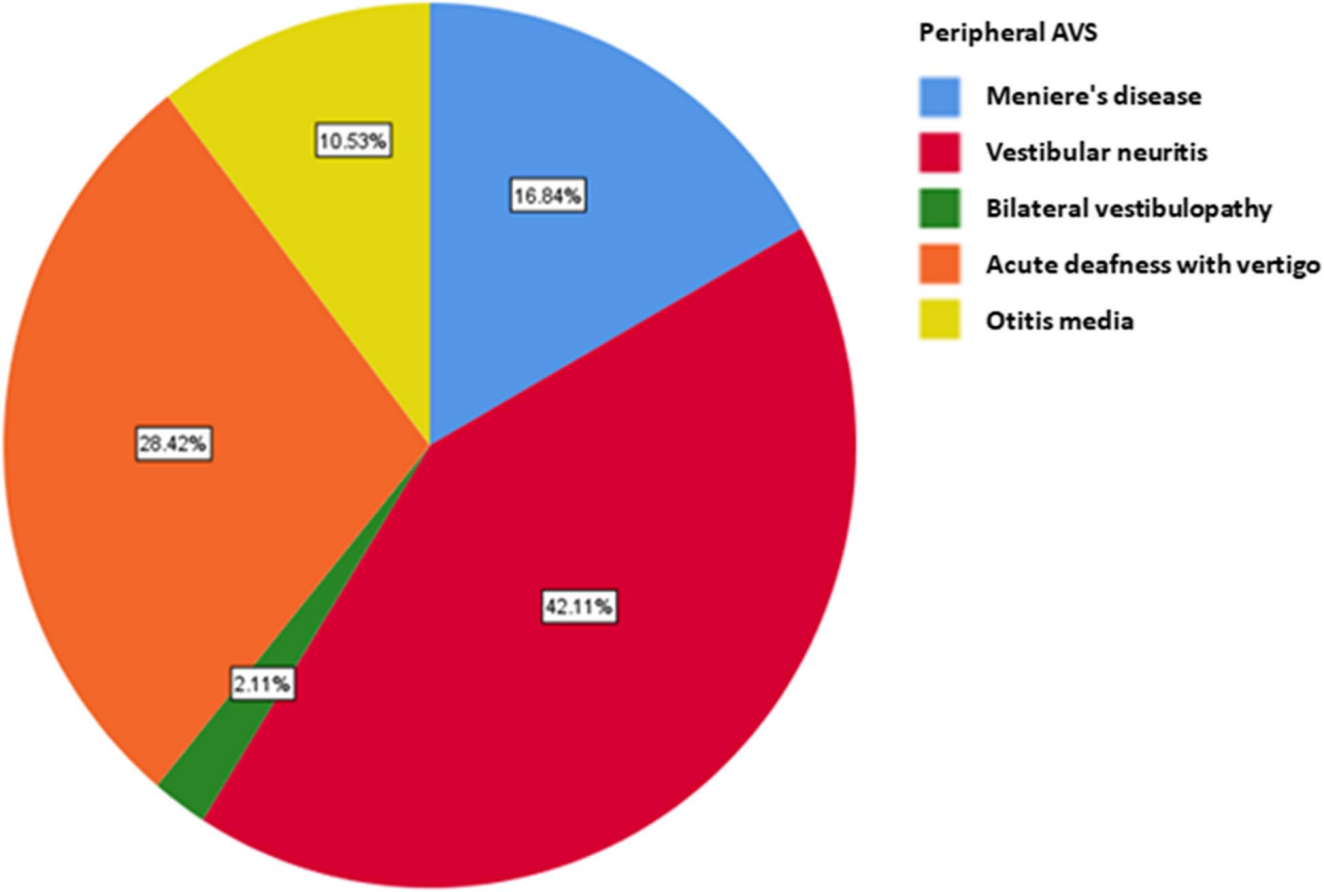
Disease spectrum of patients with peripheral AVS

In central AVS, posterior circulation cerebral infarction accounts for 46.94% and transient ischemic attack accounts for 2.04%. Vestibular migraine (36.73%) and cerebellitis (12.24%) are the second and third dominant diseases (Fig. 4). In peripheral AVS, the top three cases of peripheral vertigo are vestibular neuritis in (42.11%), acute deafness with vertigo (28.42%) and Meniere's disease (16.84%) (Fig. 5).

### Sensitivity and specificity of the HINTS

Table 2 shows that, HINTS detected 74 central AVS cases, of which 44 cases were consistent with the final diagnosis. 165 cases were diagnosed as peripheral AVS, and 160 cases were consistent with the final diagnosis. The sensitivity for diagnosing central AVS is 89.8% (95%CI: 77.0%-96.2%), the specificity is 84.2% (95%CI: 78.1%-88.9%), and the positive predictive value is 59.5% (95%CI): 47.4%-70.5%), the negative predictive value is 97.0% (95%CI: 92.7%-98.9%).

**Table 2:** Test characteristics of HINTs for AVS

HINTS Diagnosis	Final Diagnosis		Sum
	Central AVS	Peripheral AVS	
Central AVS	44	30	74
Peripheral AVS	5	160	165
Sum	49	190	239

Table 3 shows that, the sensitivity of HINTs for diagnosing cerebral infarction is 96.0% (95%CI: 77.7%-99.8%), and the specificity is 76.6% (95%CI: 70.3%-82.0%). The positive predictive value was 32.4% (95%CI): 22.3%-44.4%), and the negative predictive value was 99.4% (95% CI: 96.2%-100.0%).

**Table 3:** Test characteristics of HINTs for cerebral infarction

HINTS Diagnosis	Final Diagnosis		Sum
	Cerebral infarction	Other cases	
Cerebral infarction	24	50	74
Other cases	1	164	165
Sum	25	214	239

The sensitivity of the head impulse test for the diagnosis of central AVS is 73.5% (95%CI: 58.7%-84.6%), the specificity is 97.9% (95%CI: 94.3%-99.3%), and the positive predictive value is 90.0% (95%CI: 75.4%-96.7%), and the negative predictive value is 93.5% (95%CI: 88.8%-96.3%) (Table 4).

**Table 4:** Test characteristics of head impulse test (HIT)

Head Pulse Test	Final Diagnosis		Sum
	Central AVS	Peripheral AVS	
Central AVS	36	4	40
Peripheral AVS	13	186	199
Sum	49	190	239

The sensitivity of gaze nystagmus for the diagnosis of central AVS is 61.2% (95%CI: 46.2%-74.5%), the specificity is 92.6% (95%CI: 87.7%-95.8%), and the positive predictive value is 68.2% (95%CI: 52.3%-80.9%), the negative predictive value is 90.3% (95%CI: 85.0%-93.9%) (Table 5).

**Table 5:** Test characteristics of Gaze-evoked nystagmus (GEN)

GEN	Final Diagnosis		Sum
	Central AVS	Peripheral AVS	
Central AVS	30	14	44
Peripheral AVS	19	176	195
Sum	49	190	239

The sensitivity of TS for the diagnosis of central AVS is 26.5% (95%CI: 15.4%-41.3%), the specificity is 93.2% (95%CI: 88.3%-96.2%), and the positive predictive value is 50.0% (95%CI: 30.4%-69.6%), the negative predictive value is 83.1% (95%CI: 77.2%-87.7%) (Table 6).

**Table 6:** Test characteristics for Test of skew (TS)

TS	Final Diagnosis		Sum
	Central AVS	Peripheral AVS	
Central AVS	13	13	26
Peripheral AVS	36	177	213
Sum	49	190	239

## Discussion

### Epidemiology of acute AVS

In 1998, Hotson and Baloh proposed acute vestibular syndrome (AVS) with the characteristics of persistent vertigo, nystagmus, nausea/vomiting, head movement intolerance or poor balance [19]. It is estimated that 10%-20% of patients with dizziness in the emergency department (ED) have AVS, accounting for 25,000 to 500,000 ED visits per year in the United States alone, of which vestibular neuritis (VN) is the most common AVS etiology, with an estimated annual incidence of 3.5/100,000 [20]. Our findings are consistent with this report, showing that posterior circulation cerebral infarction and transient ischemic attach accounted for about 10% of all AVS patients [21]. Studies have shown that up to one third of strokes are missed in the initial assessment [22], since posterior circulation ischemic stroke dizziness and vertigo are very common symptoms in all patients [21]. However, since the AVS does not involve the pyramidal tract and there are no limb signs or obvious, it is difficult to distinguish it from vestibular neuritis [20]. Patients who failed to be detected will miss the best time window for thrombolysis or thrombectomy.

### Early distinction between central and peripheral AVS

Early discrimination between central and peripheral AVS is very important for early identification of possible posterior circulation stroke. CT is sensitive for hemorrhagic lesions, but not for ischemic lesions [23]. Although DWI of MRI can identify early ischemic lesions, the access to MRI examination is relatively limited [12]. Moreover, the false-negative rate of MRI diffusion-weighted imaging can still be as high as 12% 48 h after the onset of symptoms of small posterior circulation cerebral infarction [7]. The HINTS test was developed by neuro-ophthalmologists as a bedside test to rule out central causes of vertigo in patients with AVS, which has been incorporated into clinical practice by frontline emergency physicians [7, 24]. A systematic study showed that patients with a positive HINTS test had a 15-fold higher risk of developing POCS than those with a negative HINTS test, with a combined sensitivity of 95.5% and specificity of 71.2% using HINTS for any stroke. The overall positive predictive value was 59.9% and the negative predictive value was 97.2% [20]. Our study revealed a similar test performance, with less sensitivity and higher specificity. For distinguishing posterior circulation stroke, If HINTS is peripheral, the probability of posterior circulation stroke is unlikely, Patients with AICA strokes have a positive head impulse test but the nystagmus often changes direction, and large amplitude skew are helpful on central localization. Thus, any singe element of the triad that points to central HINTS requires investigation. Peripheral HINTS, does not require emergency MRI or angiography examinations, which can avoid unnecessary waste of medical resources.

A previous study showed that magnetic resonance diffusion-weighted imaging is a reasonably good method for diagnosing acute vestibular syndrome stroke, with a sensitivity of approximately 80% within the first 24 h [16]. From the data of our study, the sensitivity of HINTs is significantly higher than that of NMR. Due to the limited MRI resources in our hospital, we did not compare the early MRI within 24 h. Most of the patients with post-circulatory cerebral infarction who were diagnosed with post-circulatory cerebral infarction completed the MRI within 72 h, and some patients may be delayed to 1 week later. However, another report showed that MRI may not be sensitive to acute POCS [25], and the Oppenheim and Morita study showed that 31% of patients with vertebrobasilar ischemic stroke were falsely negative on the first DWI study within the first 24 h [26, 27].

While previous studies have suggested that up to 50% of lacunar infarcts can disappear within 48 h of symptom onset [28, 29]. Symptomatic ischemia theoretically occurs before permanent structural change displayed by MRI images. Small infarcts may result in decreased blood flow only enough to cause symptoms but not enough to cause MRI changes. This also leads to a high rate of missed diagnosis by MRI in the early stage. The study by Kattah et al. [7] showed that HINTS had higher sensitivity (100%) and specificity (96%) compared with MRI results performed within 48 h after symptom onset, with a significantly higher sensitivity and specificity than ours, which are also higher than the results of a systematic review [30]. One possible explanation is that the bedside examination of this study was performed and judged by experts in neurology.

### The rationale and performance of HINTS inspection

The neurophysiologic basis of HINTS was studied by Schmid-Priscoveanu et al., and the utilization of magnetic search coil recordings showed the loss of VOR in AVS among positive horizontal head impulse test (h-HIT) patients [31]. Additional tests potentially of diagnostic value include horizontal head-shake to examine the velocity storage mechanism in brainstem and the cerebellar nodulus [32]. The presence of horizontal directional-change or vertical post-head shaking nystagmus suggests brainstem or cerebellum abnormalities [33]. However, stroke in the AICA region remains a diagnostic challenge, as lesions affecting the vestibular root entrance, tract, or nucleus may be associated with a positive (abnormal h-HIT). To address potential localization challenges, large skew deviation (> 3 prism diopters) in AVS patients needs special attention and is a good discriminator [28]. Our study showed a sensitivity of 73.5% and a specificity of 97.9% for the head impulse (HIT) test in the diagnosis of central AVS.

Hotson et al. described the unidirectional horizontal nystagmus in peripheral vestibular lesions, following Alexander's law [19]. Kattah et al. demonstrated that nystagmus increases with fixation block in central lesions, and, therefore does not have specific localization value [34, 35]. Hotson, et al., and later Newman-Toker et al. further showed that horizontal gaze direction-change g nystagmus (GEN) as a specific hallmark of central lesions [36]. The presence of vertical or pure torsional nystagmus is also the result of central lesions. Central nystagmus symptoms are highly specific in their presentation, but unfortunately rarely present. Our study showed a sensitivity of gaze nystagmus for the diagnosis of central AVS was 61.2%, and specificity was 92.6%.

Conjugate ocular torsion test is a hallmark of abnormal otolithic ocular reflex (OOR) [35, 37]. Therefore, large deviation and ocular tilt response (OTR) are more common in central lesions [38, 39]. Although these two signs are rare in vestibular neuritis and other peripheral vestibular disorders, postoperative cases and severe, bacterial otitis media have been reported with possible exceptions [35]. Our study showed that the sensitivity of the test of skew for the diagnosis of central AVS is 26.5% and the specificity is 93.2%.

Altogether, the sensitivities of HIT, GE, and TS in the diagnosis of central AVS is 73.5%, 61.2%, and 26.5%, and the specificities were 97.9%, 92.6%, and 93.2%, respectively. After the combination of the three tests, the sensitivity was 89.8% and the specificity was 84.2%. As early as in 1988, Halmagyi and Curthoys described the head impulse test of angular vestibulo-ocular reflex, which showed a good performance [40]. However, recent studies indicate that HINTS is under-utilized in clinical practice [24, 41]. This may be because frontline providers in the emergency department have limited experience administering HINTS. On the other hand, there are other caveats for justifying the usage and reliable judgment of HINTS. For example, HINTS should only performed if patient is symptomatic at the time of vertigo onset [22, 30], and there are certain conditions when HINTS is contraindicated such as head trauma, neck trauma, an unstable spine, or neck pain [34, 35].

In contrast to previous studies in which HINTS are mainly used for the assessment of peripheral vertigo or stroke in acute continuous vertigo [24, 28], our study emphasizes the feasibility of HINTS in distinguishing central and peripheral vertigo. The data indicates that the combination of the three tests shows a good sensitivity and specificity distinguishing central and peripheral vertigo. It is also worth mentioning that false negative stroke examination by MRI is relatively out-of-dated due to the advancement of the technologies. Equipment and techniques with better magnets could provide better results. It is common for patients with central lesions to have unidirectional nystagmus as patients with peripheral lesions do. Therefore, additional signs or combinations of detections need investigation to yield higher specificity, and the head impulse test is the most important.

## Conclusions

In 1988, Halmagyi and Courthoys described the head impulse test to verify the integrity of the horizontal VOR [19]. Despite its easy bedside applicability for 34 years, and the use of the HIT in 2009 and HINTS in 2010, lack of familiarity with performance and interpretation of test results are probably responsible for under-utilization [24, 41]. To solve this obstacle from the epidemiological viewpoint, the introduction of Video-recording of the HIT and eye movements at the bedside (vHIT) show nystagmus characteristics with fixation block and identifies covert saccades [24, 31].

The positive rate of early physical examination by HINTS is higher than that of diffusion MRI. The positive results of head-impulse test are mostly associated with the peripheral AVS. Changes in gaze direction nystagmus mostly originate from central AVS. Altogether, the sensitivity of HINTS for central AVS is 89.8% and the specificity is 84.2%. HINTS are an easy-to-operate low-cost, high-sensitivity and specific examination techniques, which is easy to carry out in neurology outpatient clinics and emergency departments. It can effectively improve the diagnosis rate of cerebral infarction in AVS, and disability reduced by early diagnosis and early intervention. Since there is no specific technical equipment requirement for HINTS, and it can be widely implemented in primary hospitals after comprehensive training of the clinical practitioners.

## Data Availability

The datasets used during the present study are available from the corresponding author upon reasonable request.
